# Use of Nutraceuticals in Elderly to Fight Inflammation and Immuno-Senescence: A Randomized Case-Control Study

**DOI:** 10.3390/nu14173476

**Published:** 2022-08-24

**Authors:** Alessandro Maselli del Giudice, Ignazio La Mantia, Francesco Barbara, Silvana Ciccarone, Maria Sterpeta Ragno, Valentina de Robertis, Francesco Cariti, Michele Barbara, Luca D’Ascanio, Arianna Di Stadio

**Affiliations:** 1 Department Otorhinolaryngology, Hospital of Barletta, 76121 Barletta (BT), Italy; 2 Department GF Ingrassia, University of Catania, 95123 Catania, Italy; 3 Department Otorhinolaryngology, University Hospital of Bari, 70126 Bari, Italy; 4 Department Otorhinolaryngology, Azienda Ospeliera Riunita Marche Nord (AORMN), 61032 Fano (PU), Italy

**Keywords:** immuno-senescence, oral supplement, inflammatory cytokines, aging, zinc, vitamin D, vitamin C, nutraceuticals

## Abstract

Elderly people are at high risk of suffering from infection and being affected by severe forms of disease because their immunosystem suffers from aging. The alteration of normal immune functions causes the increase of pro-inflammatory cytokines which can expose these people to increased risk of developing pathologies as cancer, diabetes, and/or arthritis. Some supplements could be helpful for restoring normal immune functions. We conducted a case-control study to evaluate the efficacy of a supplement containing Sambucus nigra, zinc, tyndallized Lactobacillus acidophilus (HA122), arabinogalactans, vitamin D, vitamin E, and vitamin C to improve the inflammatory levels (IL-6 and CRP) and to modulate the lymphocytes growth. Additionally, we analyzed wellness by self-questionnaire. This study had two control group: a young group and an elderly one. Our study showed that treating elderly patients with the supplement for 30 days improved IL-6, CRP, and lymphocytes levels; the result was independent from the dosage of the supplements used. Elderly patients, despite the improvement, were not able to reach the same conditions of young patients; however, most of the patients (>70%) claimed to “feel better” after the use of the supplement. The use of this supplement should be considered at a low dosage for a prolonged period to reduce inflammation and modulate immune senescence in patients over 60 years old.

## 1. Introduction

Elderly people are at high risk of suffering from infection and being affected by severe forms of disease due to the aging of their immune system [1,2]; this is a multifactorial and dynamic phenomenon that affects both natural and acquired immunity and plays a critical role in most chronic diseases in older people [3].

The alteration of cell-mediated immunity in aging patients can be related to thymic involution, reduced levels of thymic hormones, and increased number of immature T lymphocytes [4]. While studies of T cell subpopulations have yielded conflicting results, it appears that T cell proliferative responses are reduced by time due to age increase. Aging is also associated with abnormalities of humoral immunity [3]. Although healthy elderly persons preserve enough functionally active neutrophils, extremely elderly people have diminished bactericidal activity and altered oxygen metabolism [4]. The studies of age-related changes in immune function are a relatively new area of investigation, which is limited by incomplete understanding of the complexities of immune mechanisms in general [5]. The aging of the immune system, called immunosenescence, determines a decrease in all immunity answers, exposing these subjects to recurrent infection and tumor [1,2,3].

In addition to the normal aging process, there are additional factors that contribute to the decrease in immune system efficiency as chronic diseases [6,7] (i.e., diabetes and hypertension), which increase the circulating oxidative species (ROS) and the poorness of the diet, potentially determining a vitamin deficiency that negatively impacts health [8]. There are specific hematological characteristics that are typical of immunosenescence: change in the ratio of CD4/CD8 (<1), increase of IL-6, neutrophilia, and high CRP levels [9,10,11,12].

Maggini et al. underlined that micronutrients and vitamins can be useful to fight the deterioration of the immune system due to aging and to reduce the inflammatory processes that are increased in the elderly [13]. Other researchers confirmed that changes in diet and bad habits and the use of supplements benefit the immune system and fight immunosenescence [14].

Vitamins can modulate inflammation and stimulate the immune answer [15,16].

By combining natural elements, it is possible to induce immune stimulation and reduction of the inflammatory elements and in this way, improving the capacity of answering to the infection [17].

We tested the immune-stimulating efficacy of an oral supplement with Sambucus nigra, zinc, tyndallized Lactobacillus acidophilus (HA122), Arabinogalactans, vitamin D, vitamin E, and vitamin C in children [18], who, like the elderly, can be affected by immune deficits. Despite a different origin of the immune deficit (incomplete maturation in children versus aging in elderly), we speculate that this oral supplement can also benefit aging people. Other studies have already shown the positive effect of vitamins and minerals to prevent chronic disease in aging [19].

This study aimed at evaluating the benefit of an oral powdered supplement (sachet) for lymphocytes, IL-6, and CRP, as well as quality of life, in elderly people compared to a control group without supplement.

The tested nutraceutical was an oral powdered supplement (Difensil^®^ IMMUNO bustine-Humana^®^ Italia S.p.A.). A single dose (1 sachet) contains the following: Sambucus nigra (183 mg), Zinc (7.5 mg), tyndallized Lactobacillus acidophilus (HA122) (1.5 × 109 cells), Arabinogalactans (15 mg), vitamin D (600 UI), vitamin E (45 mg), vitamin C (135 mg), and group B vitamins (vitamin B2: 1.4 mg; vitamin B6: 2.1 mg; vitamin B12: 2.5 mcg; folic acid: 200 mcg).

## 2. Materials and Methods

This case–control study was conducted in the Department of Otolaryngology of a tertiary hospital from September 2021 to May 2022. All procedures were approved by the local Institutional Review Board committee without release of registration number as preview by the hospital for studies using nutraceuticals and conducted in accordance with the ethical principles outlined in the Declaration of Helsinki. All participants signed a written consent accepting their enrollment in the study and authorizing the use of their data for scientific purposes.

Because we wanted to analyze the “real improvement” of the investigated parameters (lymphocytes, IL-6, and CRP), and not only the “relative gain” obtained using the nutritional supplement, but we also compared elderly patients treated with the supplement both with untreated elderly patients and with a young healthy control group. Thus, we had a young control group (YCG), an elderly control group (ECG), and two treatment groups, for a total of 4 groups.

Inclusion and exclusion criteria were defined both for elderly and young as below.

*Inclusion criteria **elderly*** (treated and untreated): people >65 hospitalized for causes other than cancer. Recruitment following SENIEUR protocol guideline [20]. *Exclusion criteria*: HIV-positive, active systemic infection, connective tissue disease, abnormal tumor marker or cancer.

*Inclusion criteria **young***: people >18 and <50. Only 1 follow-up. *Exclusion criteria*: congenital or acquired immune disease, active systemic infection, connective tissue disease, abnormal tumor marker or cancer.

Elderly patients were randomized using a computer and assigned to one of the groups. Elderly control group (ECG): untreated subjects; treatment group 1 (TG1): patients treated with one sachet of Difensil^®^ IMMUNO/die for 12 consecutive weeks; treatment group 2 (TG2): patients treated with two sachets of Difensil^®^ IMMUNO/die for 6 consecutive weeks.

In all subjects, the following blood parameters were analyzed: white cell count, interleukin-6 (IL-6), and c-reactive protein (CRP). In YCG, these parameters were collected at the baseline only (T0). In the ECG, TG1 and TG2 tests were performed at T0 (baseline), after 6 weeks with/ without treatment (T1), and after 12 weeks (T2).

### 2.1. Assay Methods

The venous blood was collected in the morning before 10 am in all subjects under fasting conditions. The anticoagulant was added to the sample and kept frozen. The serum levels of IL-6 and CRP and lymphocyte counts were detected. The levels of PCT and IL-6 were determined by the ELISA method using the human IL-6 enzyme immuno-assay kit (Abcam, Cambridge, UK), respectively. The level of CRP was determined by the immuno-agglutination method using CRP latex reagents produced by Linear Chemicals (Montagat, Barcelona, Spain). Lymphocytes were counted using the standard protocol previously described by Fenech [21].

**Figure 1:**
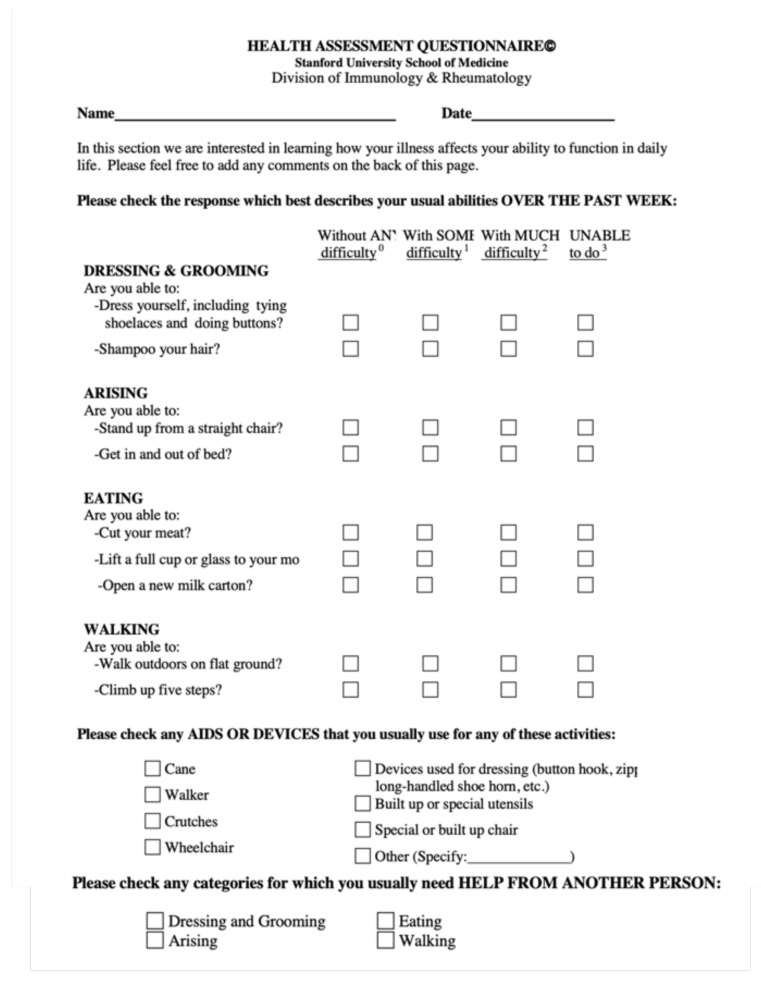
Health assessment questionnaire reproduced from Ramey DR, Fries JF, Singh G. in B. Spilker Quality of Life and Pharmacoleconomics in Clinical Trials, 2nd ed., The Health Assessment Questionnaire 1995—Status and Review. Philadelphia: Lippincott-Raven Pub., 1996, pp. 227–237 [21]. Patients answer to the question and each answer correspond to a number from 0 (normal activity) to 3 (totally uncapable to do something).

In addition, the health assessment questionnaire (HAQ) [22] was given to all subjects at T0 (Figure 1). For the elderly, the questionnaire was also given at T2.

Data about gender, age, voluptuary habits, alcohol, and smoke consumption and comorbidities were collected.

### 2.2. Statistical Analysis

One-way ANOVA was performed to compare both within and between data, and it was used to analyze the variance of lymphocyte number at the different observational points in the elderly, including the data of YCG; the same analysis was performed to analyze the variance and compare the data of IL-6 in the four groups at different follow-ups, excluding YCG at T0 only. Moreover, one-way ANOVA was also used to analyze the variances and compare differences of CRP among the four groups at the different follow up (elderly only). Ad-hoc Bonferroni–Holmes (BH) test was performed. The statistical analyses were performed with Stata^®^ and *p* < 0.05 was considered statistically significant.

## 3. Results

120 patients were included in the study; of these, 30 were in the YCG (age average 33.7 ± 8.7; 17 women and 13 men); 30 in the ECG (age average 74.3 ± 8.2; 16 women and 14 men), 30 in the TG1 (age average 73.8 ± 7.8; 17 women and 13 men) and 30 in the TG2 (age average 71.4 ± 7.9; 14 women and 16 men) (Table 1).

### 3.1. Within-Group Analysis

Table 2 shows the details about the findings we investigated, so to analyze the variances in each group at the different observational times (Table 2).

#### 3.1.1. White Cell

Patients in the ECG did not present differences compared to the number of blood lymphocytes at T0, T1, and T2 (ANOVA: *p*: 0.5).

Patients in TG1 presented statistically significant differences in the number of lymphocytes (ANOVA: *p* < 0.001) comparing T0 (baseline) and T2 (second follow-up post-treatment) (BH: *p* < 0.01); on the contrary, no statistically significant differences in the lymphocytes’ number were observed when comparing patient findings between T0 and T1 (first follow-up post treatment) (BH: *p* > 0.05). Statistically significant differences in the number of the lymphocytes were observed between T1 and T2 (BH: *p* < 0.01).

**Figure 2:**
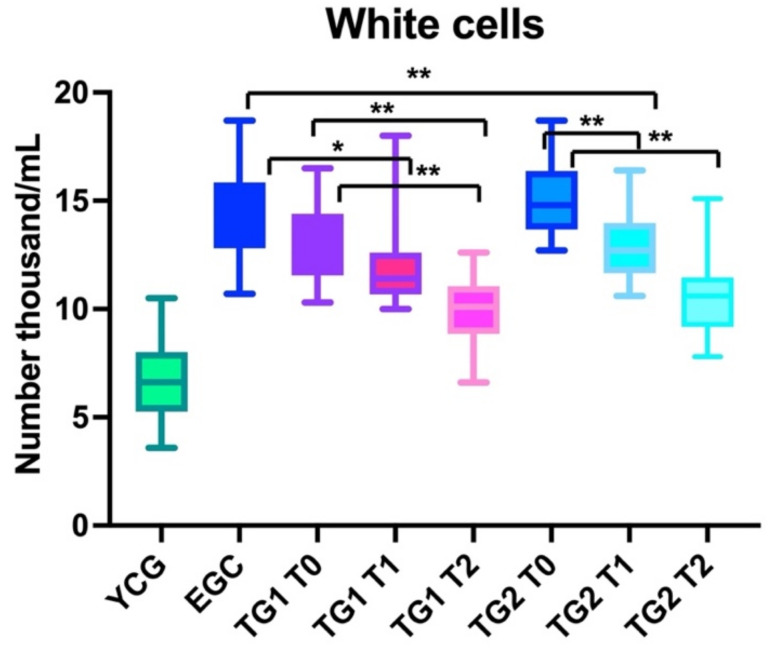
The box graph shows the differences in term of lymphocytes number between young control group (YCG), elderly control group (ECG), treatment group 1 (TG1), and treatment group 2 (TG2); for TG1 and TG2, T1 and T2 were also described. YCG and ECG were always statistically significant different from TG1 and TG2 at T1 and T2. ** *p* < 0.01 * *p* <0.05.

Patients in TG2 presented statistically significant differences in the number of lymphocytes (ANOVA: *p* < 0.001) between baseline (T0) and the first follow-up after treatment (T1) (BH: *p* < 0.05), baseline (T0) and second follow-up after treatment (T2) (II (BH: *p* < 0.01) and T1 and T2 (BH: *p* < 0.01) (Figure 2).

#### 3.1.2. IL-6

Patients in the ECG did not present statistically significant differences compared to the level of IL-6 at T0, T1, and T2 (ANOVA: *p*: 0.4)

Patients in TG1 presented statistically significant differences in the level of IL_6 (ANOVA: *p* < 0.001) when comparing baseline (T0) and the first follow-up after treatment (T1) (BH: *p* < 0.01), baseline (T0) and the second follow-up after treatment (T2) (BH: *p* < 0.01) and first (T1) and second (T2) follow-ups after treatment (BH: *p* < 0.05).

**Figure 3:**
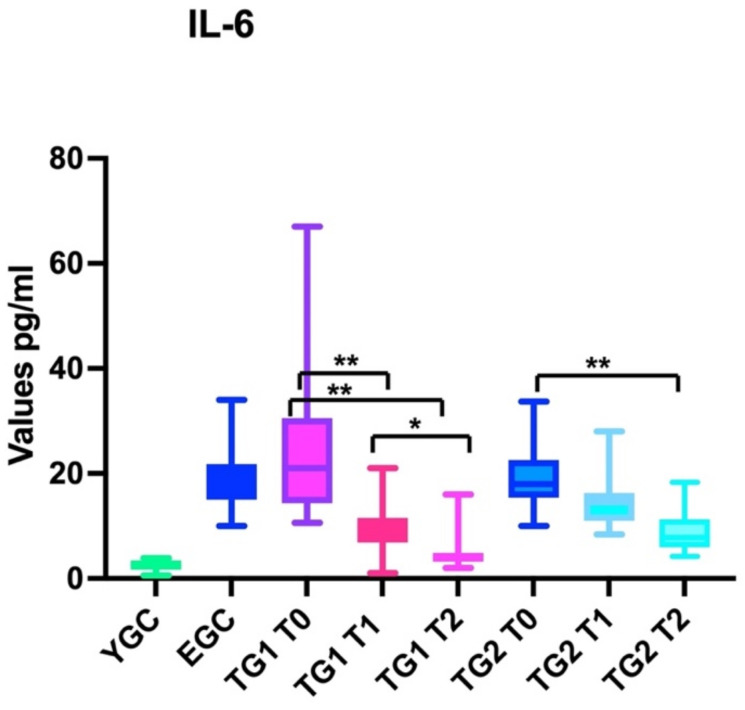
The box graph shows the differences in the IL-6 levels between young control group (YCG), elderly control group (ECG), treatment group 1 (TG1), and treatment group 2 (TG2); for TG1 and TG2, T1 and T2 were described also. YCG and ECG were always statistically significant different from TG1 and TG2 at T1 and T2. ** *p* < 0.01 * *p* < 0.05.

Patients in TG2 presented statistically significant differences (ANOVA: *p* < 0.001) in the level of IL-6 between baseline observation (T0) and the second follow-up (T2) (BH: *p* < 0.01). However, the level of IL-6 at the first follow (T1) did not sufficiently decrease to reach statistically significant value when compared with the baseline (T0) (BH: *p* > 0.05); the variance of IL-6 levels was also not enough large to observe statistically significant differences between the first (T1) and the second follow-up (T2) (BH: *p* > 0.05) (Figure 3).

#### 3.1.3. CPR

Patients in the ECG did not present statistically significant differences compared to the CPR levels at T0, T1, and T2 (ANOVA: *p*: 0.3).

Patients in TG1 obtained a statistically significant reduction of the CPR values (ANOVA: *p* < 0.001) based on the differences between baseline (T0) and the first follow-up (T1) (BH: *p* < 0.01), T0 and the second follow-up (T2) (BH: *p* < 0.01), and T1 and T2 (BH: *p* < 0.01).

**Figure 4:**
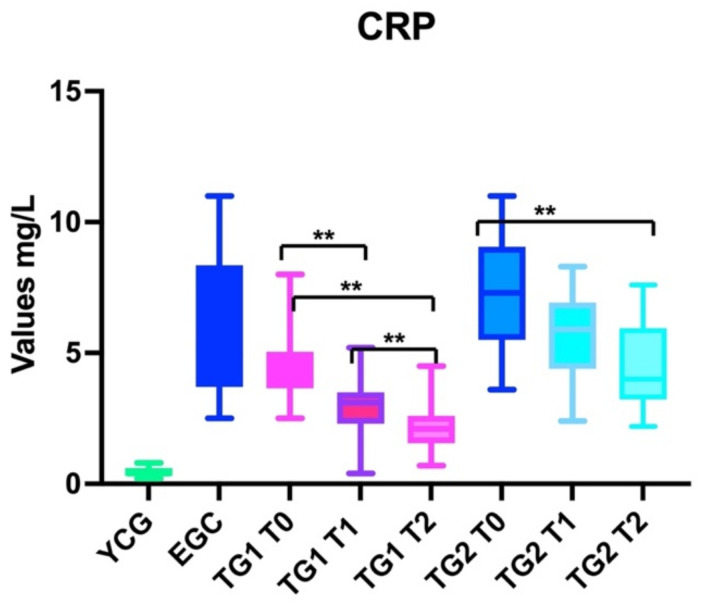
The box graph shows the differences in CRP levels between young control group (YCG), elderly control group (ECG), treatment group 1 (TG1), and treatment group 2 (TG2); for TG1 and TG2, T1 and T2 were also described. YCG and ECG were always statistically significant different from TG1 and TG2 at T1 and T2. ** *p* < 0.01.

Patients in TG2 reduced the level of CPR in a statistically significant manner (ANOVA: *p* < 0.001); specifically, we observed a statistically significant decrease of CRP comparing the baseline (T0) and the first follow-up (T1) (BH: *p* < 0.05) and T0 and the second follow-up (T2) (BH: *p* < 0.01). The reduction of CRP between the first (T1) and the second follow-up (T2) was too small to observe statistically significant differences (BH: *p* > 0.05) (Figure 4).

### 3.2. Between Groups Analysis

Table 1 shows the details about lymphocytes number and the level of interleukin-6 (IL-6) and c-reactive protein (CRP) in the young control group (YCG), the elderly control group (ECG), the treatment group 1 (TG1), and treatment group 2 (TG2) at the different observation times and the variances of these parameters among the groups (Table 1). 

#### 3.2.1. White Cells

The number of lymphocytes was statistically significantly lower when comparing the findings of YCG (ANOVA: *p* < 0.0001) and EGC at baseline (T0) (BH: *p* < 0.0001), T1(BH: *p* < 0.0001), and T2 (BH: *p* < 0.0001); moreover, the number of white cells in YCG was statistically lower than the number of the lymphocytes in TG1 at T0 (BH: *p* < 0.01), T1 (BH: *p* < 0.01), and T2 (BH: *p* < 0.01). Similar data were observed comparing YCG with TG2 at all observation points (T0, T1, and T2; *p* < 0.01).

No statistically significant differences in term of number of lymphocytes were observed comparing the findings of ECG, TG1, and TG2 at T0 (baseline) (BH: *p* > 0.05).

The number of lymphocytes after treatment was lower in TG1(BH: *p* < 0.05) and TG2 (BH: *p* < 0.01) than ECG (ANOVA: *p* < 0.0001); the white cells were less in the TG1 and TG2 groups when compared with the ECG. Similar findings were observed at T2 (BH: *p* < 0.01), both comparing TG1 and TG2.

No statistically significant differences in the number of lymphocytes were observed comparing TG1 and TG2 both at T1 and T2 (BH: *p* > 0.05) (Figure 2).

#### 3.2.2. IL-6

The level of IL-6 was statistically significantly different when comparing YCG (ANOVA: *p* < 0.0001) and EGC at T0 (BH: *p* < 0.0001), T1(BH: *p* < 0.0001), and T2 (BH: *p* < 0.0001); IL-6 was substantially lower in YGC when compared with TG1 at T0 (BH: *p* < 0.01), T1 (BH: *p* < 0.01), and T2 (BH: *p* < 0.01). Similar data were observed when comparing IL-6 of YCG with the values of TG2 at all observation points T0, T1 (BH: *p* < 0.01), and T2 (BH: *p* > 0.05).

ECG, TG1, and TG2 did not present statistically significant differences in the levels of the IL-6 at T0 (baseline) (BH: *p* > 0.05).

The levels of IL-6 were statistically significantly different when we compared the findings of EGC and TG1 and TG2 after starting the treatment (ANOVA: *p* < 0.0001); at T1, the levels of IL-6 were notably lower than the ones of ECG both in TG1 (BH: *p* < 0.01) and TG2 (BH: *p* < 0.01); the same statistically significant decrease of IL-6 was observed at T2 (BH: *p* < 0.01) when comparing the ECG with TG1 and TG2.

No statistically significant differences in the variances of IL-6 levels were observed when comparing TG1 and TG2 either at T1 or T2 (BH: *p* > 0.05) (Figure 3).

#### 3.2.3. CPR

The CRP levels were statistically significantly different (ANOVA: *p* < 0.0001) when observing these findings in YCG and EGC at T0 (BH: *p* < 0.0001), T1 (BH: *p* < 0.0001) and T2 (BH: *p* < 0.0001). The same relevant differences were observed comparing the findings of YCG and the ones of TG1 at T0 (BH: *p* < 0.01), T1 (BH: *p* < 0.01) and T2 (BH: *p* < 0.01). Similar data were observed comparing YCG with TG2 at all observation points (T0, T1, and T2 *p* < 0.01).

ECG, TG1, and TG2 did not presented statistically relevant differences in the levels of CPR at T0 (baseline) (BH: *p* > 0.05)

CRP levels varied with statistically significant differences when we compared the findings of EGC both with TG1 and TG2 after treatment (ANOVA: *p* < 0.0001); at T1, the levels of CRP were lower than the ones of ECG both in TG1 (BH: *p* < 0.05) and TG2 (BH: *p* < 0.01), and similar differences were observed at T2 (BH: *p* < 0.01) when comparing TG1 and TG2 with untreated elderly patients (ECG).

Statistically significant differences in the levels of CPR were observed comparing the findings of TG1 and TG2 at T1 (BH: *p* < 0.01) and T2 (BH: *p* < 0.01) (Figure 4).

### 3.3. Health Assessment Questionnaire Results

**Figure 5:**
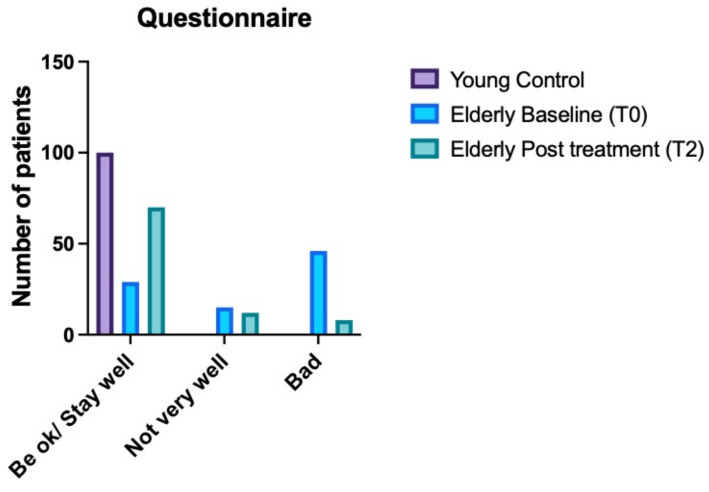
The bar graph shows the patients’ answer to the administered self-questionnaire for evaluating their health condition before and after treatment.

All YGC subjects were healthy, and they felt well (answered that they are “ok”). Among the elderly patients, 32.2% (29 subjects, including untreated and treated patients) reported that they felt well (answered “I am well”), 16.7% (15 subjects) reported that they felt “not very well”, and the remaining 51.1% (46 subjects) reported that they felt bad at the baseline (T0). At T2, 8% (8 subjects) reported that they felt bad (none in TG1 and TG2), 13.3% (12 subjects) reported feeling quite well (2 in the ECG, 4 in TG1 and 6 in TG2) and 77.7% (70 patients) reported that they have been staying well (answered that they are “ok”) (Figure 5).

## 4. Discussion

Overall, our results showed that using this nutraceutical reduced inflammation, as demonstrated by the reduction in lymphocytes (Figure 2), IL-6 (Figure 3), and CRP (Figure 4) blood levels. However, despite this improvement, elderly patients could not reach the same values of young subjects. Another interesting finding was that elderly patients in TG1 and TG2 in >70% of cases reported an improvement of their health condition (wellness) after treatment, independent of treatment group to which they belonged (Figure 5).

In comparisons between elderly untreated patients (ECG) and treated ones (TG1 and TG2), we observed that although all the patients presented the same conditions at the baseline, the patients who used the nutraceutical had better conditions than untreated ones; the benefit was observed both after 6 weeks and 12 weeks of treatment and was independent from the posology (one or two sachets par day) of the product. The two treatments used did not show any difference in term of their efficacy at T2; in fact, both TG1 and TG2 presented a reduction in lymphocyte number and the levels of IL-6 and CRP.

Patients treated with a higher dose (TG2) reduced the number of lymphocytes quicker than TG1, as shown by the statistically significant differences observed after only 6 weeks of treatment; this improvement was stable in time (statistically significant at T2) despite supplement withdrawal. Notably, the amelioration continued even if treatment was suspended (Figure 2).

The IL-6 levels decreased immediately (T1) in TG1, in which patients were treated with low dose, while it took more time to see similar important reductions (statistically significant) in TG2 (12 weeks) (Figure 3). We speculate that this was related to the better absorption of some elements of the supplement at low dosage (for example, vitamin C) that might improve the efficacy of the specific vitamin and the whole nutraceutical. In fact, excess water-soluble vitamins are excreted from the body through urine [23]; this protective mechanism may interfere with the efficacy of some supplements, especially if administered at high dosage, reducing, as in our case, the expected dose-dependent efficacy [23].

The level of CRP decreased in both treated groups after 6 weeks (T1) independently from the supplement dose (one/two sachet day); TG1 showed important decrease of CRP (statistically significant) when comparing T1 and T2; this finding could mean that by extending the time of supplement use it could possible to ulteriorly reduce the inflammatory levels (reduction of CRP). TG2 substantially reduced the CRP levels after 6 weeks, and this result remained stable after withdrawal (no statistical differences between T1 and T2) (Figure 4). These data could confirm that using the supplement for longer periods, despite at lower dosage, allows a slow, but persistent, progressive reduction of CRP.

Puzianowska-Kuźnicka et al. showed that high IL-6 and CRP levels were associated with poorer physical and cognitive performance in elderly, whereas their lower concentration was associated with better conditions [24].

Patients treated with Difensil^®^ IMMUNO reduced levels of IL-6 and CRP, which, while not reaching the values of the YCG, were notably reduced when compared with the untreated elderly (EGC); additionally, the reduction of the pro-inflammatory elements co-existed with an improvement of quality of life as evidenced by the self-questionnaire. So, despite the preliminary nature of this study and while long-term use and observation are needed to confirm our results, our data seem to agree with previous studies [13] showing that vitamins and supplements are useful for improving elderly’ health.

High concentrations of pro-inflammatory cytokines increase the risk of developing cardiovascular diseases [25], cartilage degeneration [26], lymphoproliferative disorders, multiple myeloma, osteoporosis, and Alzheimer’s disease [27]. These conditions might be prevented reducing CRP and IL-6.

The nutraceutical containing Sambucus nigra, zinc, tyndallized Lactobacillus acidophilus (HA122), Arabinogalactans, vitamin D, vitamin E, vitamin C, and group B vitamins is safe and well-tolerated, as previously shown by trials on children [18].

Several of its components can reduce the level of IL-6 as tyndallized Lactobacillus acidophilus [28], vitamin D [29], vitamin C [30], and vitamin E [31], and can contribute both to reducing CRP and lymphocyte number (vitamin D [32] and Lactobacillus acidophilus [33]). The combination of the elements generally improves immune function [34,35,36] by improving white cell function and reducing systemic inflammation [34,36].

*Sambucus nigra* reduces ROS, whose elevation contributes to worsening the inflammatory process and reduces the secretion of pro-inflammatory cytokines [37].

*Tyndallized Lactobacillus acidophilus* (HA122) modulates the immune answer, limiting the excessive production of lymphocytes [38] and reducing the levels of IL-6 [28].

*Vitamin C* reduces both CRP and IL-6 levels [30,39] as previously showed by Ellulu et al. in their human clinical trial [39] and, like Sambucus nigra, indirectly acts on inflammation by reducing ROS [30].

*Vitamin D* is widely known both for its stimulating and immune-modulating capacities [25,28]. It promotes monocyte-to-macrophage differentiation, stimulates macrophages to produce the immunosuppressant prostaglandin E2, and downregulates the expression of granulocyte–macrophage colony-stimulating factor [40]. In addition, 1.25(OH)2D3 diminishes the production by macrophages of proinflammatory cytokines and chemokines [38].

*Vitamin E* modulates T cell function by directly impacting T cell membrane integrity, signal transduction, and cell division, and indirectly through modulation of inflammatory mediators such as pro-inflammatory cytokines and prostaglandin E2 (PGE2) and the reduction of TNF-α and IL-6 [31,41].

Based on the results of this current study, we think that Difensil^®^ IMMUNO should be considered in hospitalized elderly populations as a first-line treatment because (i) it reduces the level of pro-inflammatory cytokines, (ii) it might reduce the risk of infection due to Sambucus nigra (as previously showed in children [14]), (iii) it generally improves quality of life (Figure 5).

### Study Limitations

This study presents several limitations. The first is the site of patient recruitment (hospital), and hospitalized patients could present level of lymphocytes, IL-6, and CRP higher than expected in the healthy age-matched population. Second, this population could have benefitted from the supplement because they started with higher inflammatory levels, which were more prone to decrease using anti-inflammatory molecules. Third, the limited sample size allows only preliminary results, because elderly people are affected by different comorbidities which could differently impact the results. Finally, as shown by the large SD (i.e., Figure 2), the lymphocyte count has low reliability and precision; CD4 and CD8 count would be a more accurate tool to monitor the change in the white cells [6]. For this reason, we are conducting an additional study that analyzes the variances of these lymphocytes sub-populations. Larger studies including and comparing elderly hospitalized and non-hospitalized patients, both treated with the oral supplement, are necessary to confirm the efficacy of the product on a large scale.

## 5. Conclusions

This case control study showed that a nutraceutical based on multiple synergic components like Sambucus nigra, zinc, tyndallized Lactobacillus acidophilus (HA122), arabinogalactans, vitamin D, vitamin E, vitamin C and group B vitamins (B2, B6, B12, folic acid), can reduce inflammatory levels, contain lymphocyte growth and for these characteristics could be a useful tool, especially in hospitalized patients. Patients treated with Difensil^®^ IMMUNO reduced their IL-6, and CRP levels and their lymphocyte number and improved in their wellness (self-report questionnaire). The use of the supplement at lower dosage for longer time allowed slow but consistent improvement of the observed findings; on the contrary, the higher dose for shorter time showed rapid reduction of lymphocytes number and CRP levels, but it was less efficacy on decreasing IL-6 over short time. Based on the results of this study, we suggest using this nutraceutical at low dosage for longer time to reduce inflammatory processes and the risk of developing disease strictly related to the increase of IL-6 and CRP.

Because nutraceuticals are beneficial without any adverse effects, their use should always be considered in elderly patients, even in those who apparently do not suffer from vitamin deficits. The use of these molecules as preventive treatments might reduce the onset of different diseases both related to the inflammation and immune deficit/dysfunction. Longitudinal, large scale, and longtime observational studies aiming at evaluating the beneficial effect of nutraceuticals for prevention of high-impact health system diseases should be performed.

In the future, these combinations of molecules could protect populations from severe and high-cost pathologies, prolonging life expectancy and guaranteeing good health until the end of life.

## Figures and Tables

**Table 1:** Demographic characteristics of groups.

	*Age*	*Gender*	*Hospitalized*	*Non-Hospitalized*	*Surgery Perfomed within 6 Months*	*Infection*	*Hypertension*	*Cardiac Disease*	*Obesity*	*Ipercolesterol*	*Anemia*	*Diabetes*	*Thyroid Disorders*
**YCG**	33.7 ± 8.7	17 women, 13 men	14	16	5	4	18	6	7	6	4	6	8
**ECG**	74.3 ± 8.2	16 women, 14 men	12	18	6	4	15	8	5	14	2	8	6
**TG1**	73.8 ± 7.8	17 women, 13 men	13	17	5	4	18	6	7	6	4	7	8
**TG2**	71.4 ± 7.9	14 women, 16 men	10	20	8	4	16	10	6	18	2	12	8

**Table 2:** This table shows the lymphocytes number and the level of interleukin-6 (IL-6) and c-reactive protein (CRP) in the young control group (YCG), the elderly control group (ECG), the treatment group 1 (TG1), and treatment group 2 (TG2). The *p*-values are reported in the last column of the table, and they refer to the comparison among the groups.

		YCG	ECG	TG1	TG2	*p-Value*	
*Lymphocytes*	T0	6.6 ± 1.7	13.9 ± 1.7	12.9 ± 1.7	15 ± 1.8	>0.05 (ECG. TG1 TG2)	
	T1		13.6 ±1.8	11.7 ± 1.5	13 ± 1.7	<0.001	
	T2		13.5 ± 1.5	10 ± 1.5	10.5 ± 2	<0.001	
*IL-6*	T0	2.4 ± 0.9	21.5 ± 8.6	23.4 ± 11.7	19.7 ± 6.7	>0.05 (ECG. TG1 TG2)	
	T1		21 ± 10.3	9.8 ± 4.2	14.8 ± 5.1	<0.001	
	T2		20.5 ± 11	4.8 ± 3.2	8.9 ± 3.5	<0.001	
*CRP*	T0	0.4 ± 0.1	6.1 ± 1.8	4.5 ± 1.4	7.4 ± 2.3	>0.05 (ECG. TG1 TG2)	
	T1		5.8 ±1.5	1 ± 2.8	5.5 ± 1.7	<0.01	
	T2		5.5 ± 1.4	2.1 ± 0.8	4.5 ± 1.7	<0.01	

## Data Availability

Data are available under request to the corresponding author.

## References

[1] Fuentes E., Fuentes M., Alarcón M., Palomo I. (2017). Immune System Dysfunction in the Elderly. An. Acad. Bras. Ciências.

[2] Yousefzadeh M.J., Flores R.R., Zhu Y., Schmiechen Z.C., Brooks R.W., Trussoni C.E., Cui Y., Angelini L., Lee K.-A., McGowan S.J. (2021). An aged immune system drives senescence and ageing of solid organs. Nature.

[3] Santoro A., Bientinesi E., Monti D. (2021). Immunosenescence and inflammaging in the aging process: Age-related diseases or longevity?. Ageing Res. Rev..

[4] Saltzman R.L., Peterson P.K. (1987). Immunodeficiency of the Elderly. Clin. Infect. Dis..

[5] Agarwal S., Busse P.J. (2010). Innate and adaptive immunosenescence. Ann. Allergy Asthma Immunol..

[6] Bagatini M.D., Cardoso A.M., Reschke C.R., Carvalho F.B. (2018). Immune System and Chronic Diseases 2018. J. Immunol. Res..

[7] Berbudi A., Rahmadika N., Tjahjadi A., Ruslami R. (2020). Type 2 Diabetes and its Impact on the Immune System. Curr. Diabetes Rev..

[8] Pae M., Wu D. (2017). Nutritional modulation of age-related changes in the immune system and risk of infection. Nutr. Res..

[9] Pawelec G. (2012). Hallmarks of human “immunosenescence”: Adaptation or dysregulation?. Immun. Ageing.

[10] Pawelec G. (2017). Age and immunity: What is “immunosenescence”?. Exp. Gerontol..

[11] Ferrando-Martínez S., Romero-Sánchez M.C., Solana R., Delgado J., De La Rosa R., Muñoz-Fernández M., Ruiz-Mateos E., Leal M. (2011). Thymic function failure and C-reactive protein levels are independent predictors of all-cause mortality in healthy elderly humans. AGE.

[12] Sayed N., Gao T., Tibshirani R., Hastie T., Cui L., Kuznetsova T., Rosenberg-Hasson Y., Ostan R., Monti D., Lehallier B. (2019). An Inflammatory Clock Predicts Multi-morbidity, Immunosenescence and Cardiovascular Aging in Humans. bioRxiv.

[13] Maggini S., Pierre A., Calder P.C. (2018). Immune Function and Micronutrient Requirements Change over the Life Course. Nutrients.

[14] Caruso C., Ligotti M.E., Accardi G., Aiello A., Candore G. (2022). An immunologist’s guide to immunosenescence and its treatment. Expert Rev. Clin. Immunol..

[15] Chen L., Hu C., Hood M., Zhang X., Zhang L., Kan J., Du J. (2020). A Novel Combination of Vitamin C, Curcumin and Glycyrrhizic Acid Potentially Regulates Immune and Inflammatory Response Associated with Coronavirus Infections: A Perspective from System Biology Analysis. Nutrients.

[16] Mora J.R., Iwata M., von Andrian U.H. (2008). Vitamin effects on the immune system: Vitamins A and D take centre stage. Nat. Rev. Immunol..

[17] Di Stadio A., Ishai R., Gambacorta V., Korsch F., Ricci G., Della Volpe A., Bernitsas E. (2020). Nutraceuticals as immune-stimulating therapy to fight COVID-19. Combination of elements to improve the efficacy. Eur. Rev. Med. Pharmacol. Sci..

[18] Di Stadio A., Della Volpe A., Korsch F.M., De Lucia A., Ralli M., Martines F., Ricci G. (2020). Difensil Immuno Reduces Recurrence and Severity of Tonsillitis in Children: A Randomized Controlled Trial. Nutrients.

[19] Huang H.Y., Caballero B., Chang S., Alberg A., Semba R., Schneyer C., Wilson R.F., Cheng T.Y., Prokopowicz G., Barnes G.J. (2006). Multivitamin/mineral supplements and prevention of chronic disease. Evid. Rep. Technol. Assess..

[20] Ligthart G.J., Corberand J.X., Fournier C., Galanaud P., Hijmans W., Kennes B., Müller-Hermelink H.K., Steinmann G.G. (1984). Admission criteria for immunogerontological studies in man: The senieur protocol. Mech. Ageing Dev..

[21] Fenech M., Morley A.A. (1985). Measurement of micronuclei in lymphocytes. Mutat. Res. Mutagen. Relat. Subj..

[22] Ramey D.R., Fries J.F., Singh G., Spilker B. (1996). Quality of Life and Pharmacoleconomics in Clinical Trials and The Health Assesment Questionnaire 1995—Status and Review.

[23] Shibata K., Hirose J., Fukuwatari T. (2014). Relationship between Urinary Concentrations of Nine Water-soluble Vitamins and their Vitamin Intakes in Japanese Adult Males. Nutr. Metab. Insights.

[24] Puzianowska-Kuźnicka M., Owczarz M., Wieczorowska-Tobis K., Nadrowski P., Chudek J., Slusarczyk P., Skalska A., Jonas M., Franek E., Mossakowska M. (2016). Interleukin-6 and C-reactive protein, successful aging, and mortality: The PolSenior study. Immun. Ageing.

[25] Song Y., Shen H., Schenten D., Shan P., Lee P.J., Goldstein D.R. (2012). Aging Enhances the Basal Production of IL-6 and CCL2 in Vascular Smooth Muscle Cells. Arter. Thromb. Vasc. Biol..

[26] Wiegertjes R., Thielen N., van Caam A., van Laar M., van Beuningen H., Koenders M., van Lent P., van der Kraan P., van de Loo F., Davidson E.B. (2021). Increased IL-6 receptor expression and signaling in ageing cartilage can be explained by loss of TGF-β-mediated IL-6 receptor suppression. Osteoarthr. Cartil..

[27] Ershler W.B., Keller E.T. (2000). Age-Associated Increased Interleukin-6 Gene Expression, Late-Life Diseases, and Frailty. Annu. Rev. Med..

[28] Zaharuddin L., Mokhtar N.M., Nawawi K.N.M., Ali R.A.R. (2019). A randomized double-blind placebo-controlled trial of probiotics in post-surgical colorectal cancer. BMC Gastroenterol..

[29] Labudzynskyi D., Shymanskyy I., Veliky M. (2016). Role of vitamin D3 in regulation of interleukin-6 and osteopontin expression in liver of diabetic mice. Eur. Rev. Med. Pharmacol. Sci..

[30] Bateman R.M., Sharpe M.D., Jagger J.E., Ellis C.G., Solé-Violán J., López-Rodríguez M., Herrera-Ramos E., Ruíz-Hernández J., Borderías L., Horcajada J. (2016). 36th International Symposium on Intensive Care and Emergency Medicine, Brussels, Belgium, 15–18 March 2016. Crit. Care.

[31] Huey K.A., Fiscus G., Richwine A.F., Johnson R.W., Meador B.M. (2008). In vivo vitamin E administration attenuates interleukin-6 and interleukin-1β responses to an acute inflammatory insult in mouse skeletal and cardiac muscle. Exp. Physiol..

[32] Cantorna M.T., Snyder L., Lin Y.-D., Yang L. (2015). Vitamin D and 1,25(OH)2D Regulation of T cells. Nutrients.

[33] De Simone C., Ciardi A., Grassi A., Gardini S.L., Tzantzoglou S., Trinchieri V., Moretti S., Jirillo E. (1992). Effect of *Bifidobacterium bifidum* and *Lactobacillus acidophilus* on gut mucosa and peripheral blood B lymphocytes. Immunopharmacol. Immunotoxicol..

[34] Muhamed P.K., Vadstrup S. (2014). Zinc is the most important trace element. Ugeskr. Laeger.

[35] Bergman P., Lindh A.U., Björkhem-Bergman L., Lindh J.D. (2013). Vitamin D and Respiratory Tract Infections: A Systematic Review and Meta-Analysis of Randomized Controlled Trials. PLoS ONE.

[36] Shaik-Dasthagirisaheb Y.B., Varvara G., Murmura G., Saggini A., Caraffa A., Antinolfi P., Tete’ S., Tripodi D., Conti F., Cianchetti E. (2013). Role of vitamins D, E and C in immunity and inflammation. J. Biol. Regul. Homeost. Agents.

[37] Skowrońska W., Granica S., Czerwińska M.E., Osińska E., Bazylko A. (2022). *Sambucus nigra* L. leaves inhibit TNF-α secretion by LPS-stimulated human neutrophils and strongly scavenge reactive oxygen species. J. Ethnopharmacol..

[38] Piqué N., Berlanga M., Miñana-Galbis D. (2019). Health Benefits of Heat-Killed (Tyndallized) Probiotics: An Overview. Int. J. Mol. Sci..

[39] Ellulu M.S., Rahmat A., Ismail P., Khaza’Ai H., Abed Y. (2015). Effect of vitamin C on inflammation and metabolic markers in hypertensive and/or diabetic obese adults: A randomized controlled trial. Drug Des. Dev. Ther..

[40] Guillot X., Semerano L., Saidenberg-Kermanac’H N., Falgarone G., Boissier M.-C. (2010). Vitamin D and inflammation. Jt. Bone Spine.

[41] Lewis E.D., Meydani S.N., Wu D. (2018). Regulatory role of vitamin E in the immune system and inflammation. IUBMB Life.

